# How to learn skilled communication in primary care MUS consultations: a focus group study

**DOI:** 10.1080/02813432.2021.1882088

**Published:** 2021-02-11

**Authors:** Juul Houwen, Peter L. B. J. Lucassen, Hugo W. Stappers, Karel van Spaendonck, Aniek van Duijnhoven, Tim C. olde Hartman, Sandra van Dulmen

**Affiliations:** a Department of Primary and Community Care, Radboud Institute for Health Sciences, Radboud University Medical Center, Nijmegen, The Netherlands; b Department of Primary and Community Care, Donders Institute for Brain, Cognition and Behaviour, Radboud University Medical Center, Nijmegen, The Netherlands; c Netherlands Institute for Health Services Research (NIVEL), Utrecht, The Netherlands; d Faculty of Health and Social Sciences, University of South-Eastern Norway, Drammen, Norway

**Keywords:** Medically unexplained symptoms, consultation, intervention, general practice, communication, education, focus group

## Abstract

**Background:**

Many general practitioners (GPs) experience communication problems in medically unexplained symptoms (MUS) consultations as they are insufficiently equipped with adequate communication skills or do not apply these in MUS consultations.

**Objective:**

To define the most important learnable communication elements during MUS consultations according to MUS patients, GPs, MUS experts and teachers and to explore how these elements should be taught to GPs and GP trainees.

**Methods:**

Five focus groups were conducted with homogeneous groups of MUS patients, GPs, MUS experts and teachers. MUS patients and GPs formulated a list of important communication elements. MUS experts identified from this list the most important communication elements. Teachers explored how these elements could be trained to GPs and GP trainees. Two researchers independently analysed the data applying the principles of constant comparative analysis.

**Results:**

MUS patients and GPs identified a list of important communication elements. From this list, MUS experts selected five important communication elements: (1) thorough somatic and psychosocial exploration, (2) communication with empathy, (3) creating a shared understanding of the problem, (4) providing a tangible explanation and (5) taking control. Teachers described three teaching methods for these communication elements: (1) awareness and reflection of GPs about their feelings towards MUS patients, (2) assessment of GPs’ individual needs and (3) training and supervision in daily practice.

**Conclusion:**

Teachers consider a focus on personal attitudes and needs, which should be guided by opportunities to practice and receive supervision, as the best method to teach GPs about communication in MUS consultations.

**KEY POINTS**
- Many GPs experience difficulties in communication with patients with MUS.
- There is a need to equip GPs with communication skills to manage MUS consultations more adequately.
- Role-playing with simulation patients, reflection on video-consultations and joint consultations with the supervisor may increase the GPs’ awareness of their attitude towards MUS patients and may help GPs to identify their individual learning-points.

## Introduction

Medically unexplained symptoms (MUS) are common in primary care: in about 3–11% of the presented symptoms, the general practitioner (GP) cannot attribute the symptoms to an underlying disease [1]. Like patients with psychological stress, MUS patients reflect a substantial burden on general practice [2]. GPs have a central role in the management of patients with MUS. Nevertheless, many GPs experience MUS consultations as difficult and frustrating to manage [3]. GPs indicate a lack of communication skills [4], experience difficulties in giving an acceptable explanation [5] and feel pressured to provide somatic interventions [6]. Furthermore, GPs rarely express verbal empathy [7] and explore the patients’ ideas, concerns, expectations and reasons for encounter less compared with consultations with patients with medically explained symptoms [8]. Apparently, GPs are insufficiently equipped with communication skills or have the skills but do not utilize them in MUS consultations. Therefore, there is a need to equip GPs with communication skills to manage MUS consultations more adequately.

Previous efforts to improve communication skills with MUS patients focused on changing patients’ cognitions and attributions [9]. These enhanced care interventions did not affect clinical outcome [9]. The absence of treatment effects might have been caused by patients’ resistance towards explicit psychosomatic attributions [9]. Research outside the field of MUS showed that non-specific elements such as positive communication, good quality of the doctor–patient relationship, empathy and compassion have a significant impact on patients’ health outcomes [10]. Whether this applies to the communication with MUS patients is probable but still unknown.

Given the problems of GPs in the communication with MUS patients, we consider the improvement of the communication as an important possibility as this may benefit the consultation itself. This raises the question of which communication elements GPs need in MUS consultations. We know from previous research that patients with MUS value a personalised approach in which GPs pay attention to patients’ personal circumstances, to proper somatic management of their symptoms and to good communication in which they are treated as equal partners [11]. To build further on these findings, we studied which communication elements MUS patients, GPs and MUS experts consider important and which of these elements can be learned to GPs in order to improve GPs’ communication. Furthermore, previous research found a lack of consensus amongst educators about how to teach GPs to communicate in MUS consultations, resulting in a high diversity of training programs [12]. Therefore, the second aim of this study is to learn from teachers how the identified communication elements should be taught to GPs and GP trainees.

## Methods

### Study design

We conducted five focus group interviews between May 2017 and February 2018 with MUS patients, GPs, MUS experts and teachers, respectively. We used focus groups instead of individual interviews to facilitate discussion of the participants’ views and experiences by the group discussion. Each focus group was homogeneous for the characteristics of the participants and consisted of 4–6 participants. MUS experts were defined as GPs or medical specialists with a special interest for patients with MUS and/or delivering care for specific MUS patients and/or researchers with high affinity in MUS. Teachers were defined as persons working at a university with a special interest in the field of primary care education and/or researchers with a high affinity in primary care education. A skilled moderator facilitated the discussions. Each session lasted approximately one and a half hours. We adapted the interview guide based on previous research (Appendix A) in the course of the focus group interviews. We used the COREQ guideline for the reporting of this study [13].

### Procedure

In the first and second focus group with respectively GPs and MUS patients, we explored which communication elements the participants considered relevant for MUS consultations. We compared the outcomes of the analysis of the first two focus groups (with GPs and MUS patients) with the pre-existing list with important and relevant communicational elements based on our previous research [11,14,15]. We conducted these focus groups just to be sure that the list with communication elements, based on our previous research, was exhaustive and that we did not miss communication elements. We did not find new elements. Therefore, we concluded that the list was exhaustive. We conducted 2 focus groups with experts in the field of MUS. The first one was performed with MUS experts who were mainly GPs. As we expected that medical specialists and MUS researchers have their own specific perspectives regarding communication in MUS consultations compared to GPs, we performed a second focus group with MUS experts who were medical specialists or MUS researchers. In these focus groups, we asked the MUS experts to identify from the total list (Appendix B), resulting from the previous two focus groups, the most important communication elements for MUS consultations. Furthermore, we asked them to determine which of these elements are trainable. We discussed the results of the third and fourth focus group in the fifth focus group discussion with teachers in order to explore how these elements could be trained to GPs and GP trainees.

### Study population and procedures

We approached GPs (who did not participate in our previous studies [11,14,15] in the region of Nijmegen to invite patients with MUS. Patients were identified who had in the doctor’s opinion medically unexplained symptoms and who presented their medically unexplained symptoms frequently in recent years. After consenting to participate, a researcher (JH) invited the MUS patients. Two researchers (JH or ToH) invited GPs, MUS experts and teachers by phone or by email. To obtain sufficient variation, we purposively approached participants with different backgrounds regarding sex, age, years of work experience. We invited 7 GPs of whom 5 agreed to participate in the GP focus group. Six other GPs invited 8 MUS patients for the patient focus group (4 patients agreed to participate). We invited 13 MUS experts (11 participated) and 10 teachers (6 participated), see Table 1.

**Table 1.** Characteristics of participants of all five focus groups.

	Background	Number (*n*)	Age in years (mean (min.–max.))	Sex (*n*)
Focus group 1	General practitioners	5	56 (35–64)	3 females, 2 males
Focus group 2	MUS patients	4	36 (33–38)	3 female, 1 male
Focus group 3	MUS experts	5	52 (34–63)	3 females, 2 males
Focus group 4	MUS experts	6	45 (31–58)	6 female, 0 male
Focus group 5	Teachers	6	58 (44–63)	4 females, 2 males

MUS: medically unexplained symptoms.

The mean working experience of the five general practitioners of the first focus group was 25.5 (range 2.5–35) years. The mean duration of the symptoms of MUS patients was 11 (range 5–15) years. Two patients were diagnosed with chronic fatigue syndrome, 2 with fibromyalgia while one patient had also abdominal complaints. The third focus group consisted of 3 general practitioners and 2 psychiatrists. The fourth focus group consisted of 3 MUS researchers, 1 neurologist, 1 psychiatrist and 1 psychologist.

### Data collection

An experienced male moderator (KvS, psychologist and MUS expert) moderated each focus group session, using an interview guide to direct the discussion. Before the start of the focus groups, the observer (JH) discussed the questions and main topics with the moderator. All sessions were audio-recorded and the observer took notes during the discussion. At the end of each session, the moderator summarized the discussion in order to evaluate the contribution of each of the participants and to establish whether participants agreed with the summary (member checking). After each session, the moderator and observer exchanged their preliminary impressions of the discussions. Participants were offered financial compensation for travel expenses and investment of time (€25 voucher per person).

### Data analysis

The audio-recordings of the focus group interviews were transcribed verbatim in Atlas-ti, a software program for analyzing qualitative data. Analysis of the data was performed according to the principles of constant comparative analysis. Focus group discussions and analyses proceeded iteratively. First, two researchers (JH, GP trainee and PhD student, and AvD, medical student) analysed the data from the first two focus groups. They familiarized themselves with all data by repeatedly reading all the transcripts. They coded relevant and important communication elements according to MUS patients and GPs. These codes were compared and discussed several times in consensus meetings. The findings of these focus groups resulted in a list of important communication elements. We used the findings of these focus groups together with the results of our previous studies [11,14,15] as a guide for the third and fourth focus group with MUS experts. Three researchers (JH, GP trainee and PhD student, AvD, medical student and HS, psychologist and expert in the field of education of GPs) analyzed the data. They familiarized themselves with all data by repeatedly reading all the transcripts. They coded the relevant text fragments and selected the most important communication elements and teaching methods. These codes were compared and discussed several times in consensus meetings and the final codes were applied to the transcripts. Codes referring to the same phenomenon were grouped into categories, and categories were grouped into themes. In this paper, we will only describe the results from the focus groups with MUS experts and teachers, based on the final list of important elements resulting from the first two focus groups with GPs and MUS patients. This final list of important elements, based on the results of the first two focus groups with GPs and MUS patients, is shown in Appendix B.

## Results

In the first two focus groups, no new elements emerged from the data compared to our previous studies [11,14,15]. MUS experts expanded the list with two communication elements (empowerment and meta-communication), which had not been mentioned before. From the complete list with relevant and important communication elements according to GPs and MUS patients, MUS experts identified five categories of communication elements that should be taught and trained to GPs: (1) a thorough somatic and psychosocial exploration, (2) communication with empathy, (3) creating a shared understanding of the problem, (4) providing a tangible explanation and (5) taking control. The teachers identified three teaching methods about how GPs should learn these communication elements: (1) awareness and reflection of their own feelings towards MUS patients, (2) assessment of GPs’ individual needs and (3) training and supervision in daily practice.

### What should be taught to GPs

#### Thorough somatic and psychosocial exploration

MUS experts mentioned the need to do a thorough exploration in which the GPs should ask questions to explore patients’ cognitions, ideas and concerns regarding the symptoms in order to get a complete overview of what bothers the patient. MUS experts said that the biopsychosocial model should be kept in mind. They all emphasized that patients need the opportunity to tell their whole story without being interrupted. MUS experts mentioned that listening actively and discovering verbal and non-verbal psychosocial cues (i.e. opportunities for doctors to address psychosocial issues) could help GPs to reach mutual understanding, necessary for explanation and management plan. According to MUS experts, GPs should not bring in their own views regarding the origin and management of the symptoms too quickly. First, MUS experts said that it would be better to move along with the patients’ story as this provides a better understanding of the patients’ symptoms. Further, they all emphasized the importance of an open mind without prejudgments towards the patient and regarding the origin of symptoms.

During the exploration stage, it is important to listen with an open mind. (MUS expert 4)

#### Communication with empathy

According to MUS experts, communication with empathy means that the GPs demonstrate their understanding of the perspective and feelings of the patient. But they also stated, as also noted in the literature, that many GPs experience difficulties in showing empathy, precisely in MUS consultations. However, according to MUS experts, empathy is trainable.

I don’t actually believe you can learn empathy as such. But what you can learn is to draw on your natural empathy when dealing with this group of patients. You can work on letting your capacity for empathy come freely to the fore in the consultations. (MUS expert 3)

When GPs experience difficulties to empathise with the patient’s view regarding the origin of symptoms, MUS experts said that GPs should try to focus on the patient’s suffering.

But I think empathy, or understanding for the burden this brings, can be a good starting point for progress in a conversation. Rather than saying that’s weird, that can’t happen and then having to explain why mobile phone masts can’t be the cause. You’re better off saying: Wow, that must be so difficult because every time you walk out of the door here, there’s that mast again, what happens then? I think that would get a conversation going. (MUS expert 11)

According to MUS experts, GPs should be aware of their own attitudes and feelings towards MUS patients. MUS experts stated that self-reflection might help GPs to be more aware of their own attitude towards MUS patients which may help them to empathize with the patient and to improve interaction with the patient.

If you can’t accept the idea that radiation could cause complaints, you include that point in the consultation. And then you’ll probably be inclined to rebut that idea, whereas that’s exactly what you shouldn’t be trying to do. You do indeed need to find some understanding. And it helps you as well. Be aware of your own preconceptions and everything you bring with you into that consultation and how that affects the interaction between you and the patient. (MUS expert 6)

#### Shared understanding of the problem

MUS experts mentioned the importance of a shared understanding of the problem, defining this as finding common ground about the patients’ problem (i.e. bringing together the GPs’ and patients’ viewpoint about the nature of the patients’ problem). A shared understanding of the problem is also necessary to be able to give an explanation and to develop a treatment plan.

I think a shared definition of the problem is crucially important. If you can’t agree on exactly what the problem is, it makes all the rest very tricky. Then you can explain things or draw up a treatment plan until you’re blue in the face but none of it will work. (MUS expert 1)

According to MUS experts GPs can only attain a shared understanding through exploration, being empathic, listening actively to discover psychosocial cues and being aware not to keep thinking in their own concepts or follow their own agenda.

I think we’re very quick to figure out what is going on and then we basically want to define and present the problem as we see it. But we should hold back and wait, and only start a cautious process of drawing out information when you think we’ve now really explored everything that we need to. These are skills that you can learn. (MUS expert 1)

MUS experts stated that it is important to know that the nature of the patient’s problem is not always about reducing the severity of symptoms, but may also be about other aspects, such as problems in functioning, cognitions or emotions.

#### A tangible explanation

MUS experts mentioned the importance of giving an acceptable explanation delivered at a time when the patient is ready for it. To ensure that the patient is ready for it, MUS experts said that GPs should focus on the physician–patient relationship and thorough exploration. Giving an explanation too quickly frequently results in the patient rejecting it.

Then eventually the penny drops, or they accept something. Then you can finally present that model again that you’ve already suggested several times only to have it resolutely rejected. Then they’re ready for it and that’s a magical moment. And to get them ready for it, you need to keep on exploring and stay in contact. (MUS expert 2)

MUS experts said that GPs should use explanation models in which they involve bodily elements such as physiology, the disturbance of the stress system, the role of the hormone system and the function of the immune system. Further, some MUS experts said that GPs should use information from the physical examination within their explanation. Other MUS experts recommended connecting patients’ life events with the disturbance of their stress system and their current symptoms.

There are actually some models for MUS that you could use to explain quite a lot of the things patients experience. Personally, I like the allostasis model. It’s a model where you basically make links between the things people go through in their lives, the disruption of the stress system and a whole range of complaints that they accumulate in the course of their lives as a result. Right, if you can offer this as a model without being too prescriptive, my experience is that patients really feel like you are acknowledging them. (MUS expert 4)

Further, MUS experts said that GPs should use their medical authority and expertise in their explanation. As an example, they could say that MUS are common in primary care, they have a lot of experience in managing MUS and that most of the symptoms are self-limiting.

#### Taking control

According to MUS experts, taking control means that both the GP and the patient exert control over the consultation. The GP should guide MUS patients over the whole care process, whereby (s)he can play a crucial role in the management of these patients.

Being in control covers the entire process of assisting someone with MUS because there are so many aspects of the healthcare system where MUS patients can get stuck. What strikes me about patients consulting for MUS is that they’re also getting nowhere in the healthcare system anymore. That is the control you can offer as a GP. Looking with them at where they are now and seeing what their next step is. (MUS expert 3)

As many GPs experience MUS consultations as chaotic, GPs should learn to take control during the consultation concerning the process. GPs should particularly pay more attention to the structure of the consultations by defining the different stages of the consultation more clearly and making more frequent use of summaries.

According to MUS experts, patients can take control as well by discussing their own goals. The patient’s goals should have priority over the GP’s goals.

Rather than taking control yourself, where you as the doctor aim to achieve all kinds of things, sometimes you should make sure you put the patient in control. I think you need to make that distinction, because you can do all kinds of things that patient doesn’t want and that isn’t going to work. (MUS expert 5)

### How should this be taught

The teachers agreed with the importance of the five communicational elements as described above. They mentioned three teaching methods for these elements: (1) awareness and reflection on their own feelings towards MUS patients, (2) assessment of GPs’ individual needs and (3) training and supervision in daily practice.

#### Awareness and reflection on their own feelings towards MUS patients

Teachers said that GPs should be taught to be more aware of their own attitudes and feelings towards MUS patients. GPs should reflect on their attitude and feelings in daily MUS consultations. Role-playing with simulation patients was mentioned as a method to increase the GPs’ awareness of their attitude and feelings towards MUS patients. In a role-play, GPs are invited to reflect on a specific moment in which they experience problems with their attitude.

If you’re thinking of an educational intervention, I think you only see the need when you are in that situation yourself. So you can reflect on ‘what do I do then’ if you adopt, as it were, one such patient in your GP traineeship who you follow throughout your traineeship. Then I think you can reflect after a consultation on ‘how did I feel, what effect did it have on me and what did I do and what didn’t I do’. (Education expert 6)

Teachers also suggested joint consultations, in which GP residents perform consultations with MUS patients together with their supervisor, with the aim to share and discuss their feelings and experiences.

I’m thinking more and more that you should actually start a MUS process with your supervisor, for example. Never do it alone but always with your supervisor. And then you can discuss your feelings together. (Education expert 7)

#### Assessment of GPs’ individual needs

Education should be based on identified individual needs. Therefore, teachers stated, GPs should identify their own communication barriers in MUS consultations resulting from real-life consultations. In order to fit well with these needs, teachers said that GPs should work in small groups.

So you need to take what that doctor comes up against in practice (and they all come up against it) and translate that into the educational situation and then key in as a teacher to those individual people’s experiences. That means you probably need to work in relatively small groups, as that’s one of the more important educational principles. (Education expert 8)

Further, one teacher mentioned role play with simulation patients to identify individual learning points. According to them, the feedback of either the simulation patient or other GPs may help GPs identify their personal needs.

#### Training and supervision in daily practice

GPs should create conditions in daily practice to practice and reflect on their own MUS consultations. As an example, they mentioned the use of video consultations, which stimulate GPs to think about what they should have done in the video consultation. Training with real patients or simulated patients, feedback by trainers and observing role-models are important methods.

They are learning, they go back to the workplace and hopefully they see the same patient again. Then you practice again and again. So it’s not a one-off thing: it’s a longitudinal process that involves experiences in real situations, with video recordings. And I think you could even have individual feedback. (Education expert 8)

## Discussion

### Summary of main findings

The most important communication elements during MUS consultations which should be improved according to GPs, MUS patients and MUS experts are: (1) a thorough somatic and psychosocial exploration, (2) communication with empathy, (3) creating a shared understanding of the problem, (4) providing a tangible explanation and (5) taking control. Teachers considered the following methods as appropriate for teaching the communication elements to GPs or GP trainees: (1) stimulating awareness and reflection of GPs about their feelings towards MUS patients (2) assessment of GPs’ individual needs and (3) training and supervision in daily practice.

### Comparison with existing literature

Our findings of a thorough exploration [16–21], communication with empathy [17–19] a shared understanding of the patients’ problem [17–19], giving a tangible explanation [16–21] and shared control (19–21) have been reported before in the context of MUS and are thus far from new. This raises the question of whether GPs really use these elements in consultations and why so many GPs experience MUS consultations as challenging and feel powerless during these consultations. There are indications that GPs do not use these elements in consultations with severe MUS. Or, GPs try to use the elements but do this in a negative atmosphere. The common factor here might be the negative attitude towards patients with unexplained symptoms which is already present in medical students after a few years of education. This negative attitude may be the consequence of the predominance of the biomedical model in medical education. MUS, almost by definition, do not fit into a biomedical model. MUS is therefore being perceived as complex and many students and educators struggle to understand MUS due to the ambiguity surrounding the cause. The curriculum is focused on explainable diseases, which are less complex to learn. Therefore, many students are not equipped with adequate knowledge and communication skills, leading to ineffective doctor–patient communication. This may result in negative experiences of patients and the persistence of symptoms which may enhance the negative attitude of physicians further. Furthermore, MUS are often associated with negative stereotypes, also contributing to a negative attitude. The experience of MUS as complex, the low priority of teaching MUS, the negative attitudes of tutors, the low prestige of MUS in the hierarchy of medical problems and the opinion of physicians about the relative unimportance of MUS cause barriers for the implementation of education about MUS in the medical curriculum [22–25]. Instead of being focussed on a biomedical model, medical education should focus on a broader conceptualization of illness within a biopsychosocial model and should focus on the illness experience of patients. The biopsychosocial model assumes that the symptoms presented by patients always have somatic, cognitive, emotional, social and behavioural dimensions and that the experience of symptoms takes place in constant interaction with the environment. The model is certainly fruitful for the management of MUS. Further, a focus on the implementation of teaching methods about MUS may miss the point when we do not consider the attitudes of GPs and GP trainees concerning MUS. This could also mean that a change of focus on MUS during medical education is the most important condition for improving the care for MUS patients. In this respect, it is important to know that previous research showed that a seminar about MUS was associated with a more favorable attitude towards MUS [22]. Although we consider a change of attitude as most important, improvement of the GPs’ repertoire of communication skills is important to be the channel to express one’s attitude. An important example here is teaching GPs and trainees how to provide a clear and tangible explanation of the unexplained symptom. Many GPs have problems with explaining MUS to patients, which is completely understandable in light of the lack of attention for this topic during medical education.

Further, MUS experts said that GPs should not give an explanation too quickly as patients may reject this. When GPs label symptoms as medically unexplained, it is important that GPs do not miss a somatic underlying disease. However, Eikelboom et al. described in a review that the percentage of misdiagnoses in patients with MUS was relatively small [26]. Furthermore, Houwen et al. analysed when and how GPs recognised MUS [27]. They found that GPs labelled symptoms as medically unexplained soon after the start of the consultation and that GPs clearly pointed out what triggered them in their labelling process. This suggests that GPs do not experience uncertainty about missing a diagnosis.

Malterud et al. state that in primary care there is more in diagnostic work than hypothesis testing and pattern recognition [28]. Apart from these more or less objective actions, they address the importance of interpretive work which is inherently subjective. The interpretive actions from the GP are based on information such as knowing the person for a long time and, consequently, being familiar with the person’s appearance or verbal utterances. The GP may transform this information into questions for understanding, thus giving access to an alternative understanding of the problem. This approach may have the potential for the implementation of education about MUS.

Furthermore, Salmon et al. described that applying communication skills is not necessarily good communication because communication is always subjectively shaped [29]. The concept of more or less objectively defined communication skills is inherently reductionistic. The danger of identifying communication elements as ‘skills’ is that they come to define good communication even when there is no evidence of benefit for patients. Salmon proposed that skilled communication should incorporate patients’ individuality and that practitioners should be trained in flexibility and creativity in communication [29]. Therefore, learners should develop the capacity to use the elements described in our study flexibly and, if necessary, to refrain from them depending on the situation.

### Strengths and limitations

This study has a number of strengths. First, participants with several backgrounds (GPs, MUS patients, MUS experts, teachers) participated which provided insights from different fields. Second, by comparing the analysis of the first two focus groups (with GPs and MUS patients) with the pre-existing list with important and relevant communicational elements based on previous research [11,15], we concluded that the list was exhaustive. However, MUS experts expanded the list with two communication elements (empowerment and meta-communication), which had not been mentioned before. Third, we used a qualitative approach with a cyclical process of gathering information and analysis and analysis performed independently by two researchers. This study has also some limitations. We performed only two focus groups with MUS experts. Although we expected that medical specialists and MUS researchers had their own specific perspective regarding communication in MUS consultations, we did not find differences with the results of the first focus group. Further, we did not match each communication element with a specific learning method as the teachers were more focused on overarching teaching methods rather than just focussing on each communication element separately. Another possible limitation could be the majority of females in all focus groups. To obtain sufficient variation of the data, we purposively approached participants with different backgrounds regarding age, clinical background, sex. Although the majority of the participants were women, we do not expect that a different distribution of sex would have led to different conclusions as we did not find new themes.

### Implications for further research and daily practice

This study gave more insight on which important communication elements should be taught to GPs and GP trainees, and how these elements can be trained. The next step will be to develop a communication training tool with the five elements found in this study. The tool should be acceptable for patients with MUS and feasible in daily general practice. The focus of this tool should be on attitude, needs assessment and training with supervision.

## Conclusion

MUS experts identified five categories of communication elements that should be taught and trained to GPs: (1) a thorough somatic and psychosocial exploration, (2) communication with empathy, (3) creating a shared understanding of the problem, (4) providing a tangible explanation and (5) taking control. Role-playing with simulation patients, reflection on video-consultations and joint consultations with the supervisor may increase the GPs’ awareness of their attitude towards MUS patients and may help GPs to identify their individual learning-points.

## Data Availability

Additional data can be accessed *via* Radboudumc, department of primary and community care.

## Appendix A

***Interview guide focus group general practitioners (GPs)***

How can a GP make sure that he/she:

1. Takes care of a good preparation of the consultation?
2. Matches his/her agenda with the patients’ agenda?
3. Structures the consultation?
4. Takes control the consultation?
5. Shows empathy during the consultation?
6. Shows an open attitude without to be prejudiced?
7. Explores the complaints in depth, picks up cues and explores these?
8. Formulates an explanation and recognizes his/her limits in explaining the cause of the complaints?
9. Comes up with a plan?

***Interview guide focus group MUS patients***

1. When you visit your general practitioner (GP) for medically unexplained symptoms, what do you expect from your GP?
2. Do you have the impression that your GP expects something from you too?
3. Which GPs’ behavior or attitude makes you feel being helped? And why?
4. On contrary, which GPs’ behavior or attitude makes you not feel being helped? And why?

***Interview guide focus group MUS experts***

1. Which of the communication elements as listed below (Appendix B) are the most important, can be trained to GPs and can be incorporated in MUS consultation (in primary care)? (Select the three most important elements)
2. Which of the communication elements as listed below (Appendix B) are the least important, cannot be trained to GPs and cannot be incorporated in MUS consultation (in primary care)? (Select the three least important elements)

***Interview guide focus group teachers***

1. How can we teach the next 5 communication elements to GPs: (1) a thorough somatic and psychosocial exploration, (2) communication with empathy, (3) creating a shared understanding of the problem, (4) providing a tangible explanation and (5) taking control. The five communication elements have been described in detail as in the result section.
2. Which training methods are useful to teach this?
3. Are there already pre-existing methods to teach GPs the 5 communicational elements?

## Appendix B

***Complete list of identified important and relevant communication elements***

Knowing the person/Empathy/Open and approachable/Dialogue/Time and space/Clarity/Equality/Quiet atmosphere/Shared problem definition/Shared decision making/Exploration/Identification of cause/Explanation/Structuring/Reassurance/Following the patient/Take charge/Non-verbal behavior/Connecting somatic and psychological symptoms/Create self-awareness/Match with patient’s agenda/Avoid giving the patient an unpleasant feeling/Avoid being prejudiced/Preparation of the consultation/Acknowledge uncertainty about the origin of the symptoms/Offer a specific management plan.
